# Mechanism of *Astragalus membranaceus* (Huangqi, HQ) for treatment of heart failure based on network pharmacology and molecular docking

**DOI:** 10.1111/jcmm.18331

**Published:** 2024-05-23

**Authors:** Qiuxiang Chen, Juan Wang, Lihua Sun, Bayinsilema Ba, Difei Shen

**Affiliations:** ^1^ Department of Cardiology, Renmin Hospital of Wuhan University, Cardiovascular Research Institute of Wuhan University Hubei Key Laboratory of Cardiology Wuhan China; ^2^ Department of Neurology Renmin Hospital of Wuhan University Wuhan China; ^3^ Department of Cardiology The Fifth Affiliated Hospital of Xinjiang medical University Urumchi China

**Keywords:** *Astragalus membranaceus* (Huangqi, HQ), heart failure, molecular docking, network pharmacology, traditional Chinese medicine (TCM)

## Abstract

Heart failure is a leading cause of death in the elderly. Traditional Chinese medicine, a verified alternative therapeutic regimen, has been used to treat heart failure, which is less expensive and has fewer adverse effects. In this study, a total of 15 active ingredients of *Astragalus membranaceus* (Huangqi, HQ) were obtained; among them, Isorhamnetin, Quercetin, Calycosin, Formononetin, and Kaempferol were found to be linked to heart failure. Ang II significantly enlarged the cell size of cardiomyocytes, which could be partially reduced by Quercetin, Isorhamnetin, Calycosin, Kaempferol, or Formononetin. Ang II significantly up‐regulated ANP, BNP, β‐MHC, and CTGF expressions, whereas Quercetin, Isorhamnetin, Calycosin, Kaempferol or Formononetin treatment partially downregulated ANP, BNP, β‐MHC and CTGF expressions. Five active ingredients of HQ attenuated inflammation in Ang II‐induced cardiomyocytes by inhibiting the levels of TNF‐α, IL‐1β, IL‐18 and IL‐6. Molecular docking shows Isorhamnetin, Quercetin, Calycosin, Formononetin and Kaempferol can bind with its target protein ESR1 in a good bond by intermolecular force. Quercetin, Calycosin, Kaempferol or Formononetin treatment promoted the expression levels of ESR1 and phosphorylated ESR1 in Ang II‐stimulated cardiomyocytes; however, Isorhamnetin treatment had no effect on ESR1 and phosphorylated ESR1 expression levels. In conclusion, our results comprehensively illustrated the bioactives, potential targets, and molecular mechanism of HQ against heart failure. Isorhamnetin, Quercetin, Calycosin, Formononetin and Kaempferol might be the primary active ingredients of HQ, dominating its cardioprotective effects against heart failure through regulating ESR1 expression, which provided a basis for the clinical application of HQ to regulate cardiac hypertrophy and heart failure.

## 1 INTRODUCTION

Heart failure represents a growing chronic medical condition with major implications on patient morbidity, mortality and cost to health care systems.^1^ Treatment for heart failure depends on its cause, symptoms, and ejection fraction, a measure of the heart's squeezing function.^2^ Moreover, heart failure is a complication of oncological treatments that may have dramatic clinical impact.^3^ Cardiotoxicity is a major side effect of the chemotherapeutic drug doxorubicin or sunitinib or trastuzumab.^4^ Hence, development of new available nutraceuticals to manage chemotherapy‐induced cardiotoxicity is receiving attention.^5^ The onset of heart failure is typically preceded by cardiac hypertrophy, a response of the heart to increased workload, a cardiac insult such as a heart attack or genetic mutation. Cardiac hypertrophy is usually characterized by an increase in cardiomyocyte size and thickening of ventricular walls. Initially, such growth is an adaptive response to maintain cardiac function; however, in settings of sustained stress and as time progresses, these changes become maladaptive and the heart ultimately fails.^6^ Heart failure frequently is the unfavourable outcome of pathological heart hypertrophy.^7^ Nevertheless, because of the unknown potential molecular mechanisms, the lack of effective approaches for preventing cardiac hypertrophy or heart failure remains a plague to patients and clinicians.

Traditional chinese medicine (TCM), a verified alternative therapeutic regimen, has been used to treat heart failure, which is less expensive and has fewer adverse effects. In TCM clinical practice, combining illness with syndrome is a key therapy principle. The primary six syndromes in heart failure patients are deficiency of Yang, Qi, blood stasis, fluid retention, Yin deficiency and phlegm turbidity.^8^, ^9^ The major syndrome ingredient of cardiac disorders is discovered to be blood stasis, followed by Qi deficiency.^10^ The primary malfunction in heart failure is a long‐term heart‐Qi and heart‐Yang deficiency, leading to blood circulation stasis.^11^ As a result, it refers to the therapeutic principles of benefiting heart‐Qi and warming heart‐Yang collectively, especially through increasing blood circulation to dispel blood stagnation and remove oedema. *Astragalus membranaceus* (Radix astragali, Huangqi, HQ) is a regularly recommended TCM herb for treating various heart failure symptoms.^12^ For instance, Feng et al. demonstrated that Astragaloside IV, a principle active constituent of HQ, alleviates heart failure by modulating Nrf‐2.^13^ Ma et al. reported that Astragalus polysaccharide, a key active ingredient isolated from HQ, prevents heart failure‐induced cachexia by alleviating excessive adipose expenditure in white and brown adipose tissue.^14^ HQ injection, which is a formulation of an extract of HQ, is a most commonly used type of proprietary Chinese medicine for the clinical operation of treating chronic heart failure. There were 62 randomized controlled trials (RCTs) and quasi‐RCTs evaluated.^15^ However, the trial procedures were of poor quality, and the existing research were insufficient to demonstrate the effectiveness and safety of HQ injection. Identifying the primary active constituents, particular activities of these active compounds, and underlying molecular mechanisms may help to expand HQ's application in treating cardiomyocyte hypertrophy and heart failure.

In consideration of the complicated function process and mechanism of TCM herbs, in this study, major active ingredients of HQ were focused and Isorhamnetin, Quercetin, Calycosin, Formononetin and Kaempferol were investigated. Isorhamnetin widely exists in several TCM herbs, such as HQ, Hippophae rhamnoides L.,^16^ Ginkgo biloba L.^17^ and Semen Lepidii.^18^ Isorhamnetin has been widely recognized for its anti‐inflammatory, antioxidative, antiadipogenic, antiproliferative and antitumor properties; moreover, Isorhamnetin protects against cardiac hypertrophy,^16^ cardiomyopathy,^17^ and Ang II‐induced fibrosis and hypertrophy in the myocardium of mice.^19^ Quercetin is a flavonoid that is abundant in nature. According to pharmacological research, quercetin can postpone vascular endothelial functional deterioration and cardiac terminal damage.^20^ Quercetin can also modulate islet function and have a regulatory role in the prevention of cardiac fibrosis.^21^ Calycosin, as one of the quality markers of Qiliqiangxin capsule, participates in Qiliqiangxin capsule treatment of chronic heart failure.^22^, ^23^ Formononetin is another quality marker of Qiliqiangxin capsule.^22^, ^24^ Furthermore, Formononetin exerts a cardioprotective effect in doxorubicin‐induced chronic heart failure model rats.^25^ Kaempferol also has a wide range of pharmacological activities, such as anti‐oxidant, anti‐inflammatory, anti‐microbial, anti‐diabetic, and anti‐tumour properties.^26^, ^27^ For example, Kaempferol protects against Ang II‐induced cardiac remodelling by relieving inflammation and oxidative stress induced by Ang II.^28^

TCM's pharmacodynamic foundation and mechanism are challenging to understand due to its multi‐compound, multi‐pathway, and multitarget properties. Hopkins, a British pharmacologist, suggested network pharmacology for the first time in 2007^29^ to understand the biological mechanism of pharmacological intervention in illness. The network pharmacology mechanism of TCM research is consistent with TCM's overall function, and the network pharmacology approach is reliable and dependable.^29^ The present study retrieved active compounds of HQ, compared drug‐ and disease‐target genes, employed network pharmacology to build an ingredient‐signalling‐target network, and investigated the specific effects and molecular mechanism of the main active ingredients on Ang II‐induced cardiomyocyte hypertrophy, laying the groundwork for future research on HQ's mechanism of action in the treatment of cardiac hypertrophy and heart failure.

## 2 MATERIALS AND METHODS

### 2.1 Active compounds of HQ retrieved from Traditional Chinese Medicine System Pharmacology Database (TCMSP)

The active compounds of HQ were obtained from TCMSP (http://lsp.nwu.edu.cn/tcmsp.php).^30^ Using pharmacokinetic information retrieval filters, active ingredients' information was retrieved based on ADME (absorption, distribution, metabolism and excretion). The screening conditions have criteria of OB ≥40% and DL ≥0.2.

### 2.2 Information of HQ active compounds

The molecular structure, biological activity and related targets of HQ active compounds obtained from the last step were obtained from NPASS website (http://bidd.group/NPASS/).^31^ The drug targets of the active ingredients in HQ were collected from the PubChem database (https://pubchem.ncbi.nlm.nih.gov/).

### 2.3 Disease target retrieval through differentially expressed genes analysis

GSE25765 contains differentially expressed genes (DEGs) between 6 failing heart tissues and 6 control heart tissues. RNA extraction and hybridization were performed on Affymetrix microarrays. Individual *p*‐values were computed, and the false discovery rate (FDR) for numerous testing correction was computed using the Benjamini and Hochberg method. DEGs were considered using the threshold of |logFC| > 1, *p* < 0.05. Drug targets and DEGs were compared and 391 overlapped genes were selected for following analyses.

### 2.4 Kyoto Encyclopaedia of Genes and Genomes (KEGG) signalling pathway enrichment annotation

KEGG Mapper (https://www.genome.jp/kegg/mapper/)^32^ was exploited to perform signalling pathway enrichment annotation.

### 2.5 Construction and analysis of networks

Cytoscape_v3.8.0 was used to construct the compound‐target‐pathway networks.^33^ Nodes represented the ingredients, proteins, or pathways within these graphical networks, while edges represented the ingredient‐target or target‐pathway interaction. Based on the online tool STRING database, drug genes were analysed, and a protein interaction network was constructed with the thresholds of the interaction score <0.9 were screened.

### 2.6 Prediction of the binding between HQ active ingredients and target genes by AutoDock

The AutoDock program has been widely used to predict the docking of small molecule ligands with large molecule receptors,^34^ and is characterized by multiple advantages such as high accuracy, fast speed, and no charge. The binding between ligand and receptor molecules was primarily evaluated via the evaluation of the binding energy in AutoDock. Specifically, the lower binding energy between the protein receptor and small molecule ligand suggested a better binding between the two, thus indicating a greater possibility of interaction. The protein structure of ESR1 (PDBID: 7jkw) was download based on PDB database (https://www.rcsb.org/). The 3D crystal structures of celastrol, Formononetin, Isorhamnetin, Kaempferol, Quercetin were download from PubChem database. Molecular docking was applied through AutoDockTools‐1.5.6 to verify the correlation between the key targets and the active compounds.

### 2.7 Cell lineage and cell culture, treatment

Rat cardiomytocyte cell line H9c2 was procured from ATCC (CRL‐1446, Manassas, VA, USA) and cultivated in Dulbecco's Modified Eagle's Medium (DMEM, 30–2002, ATCC) added with 10% Fetal Bovine Serum (FBS, 30–2020, ATCC). Cells were cultured at 37°C in a humidified atmosphere containing 5% CO_2_. The H92c cells were co‐treated with 1 μM recombinant Ang II and 5 active compounds for 24 h respectively according to previous researches (20 μM Quercetin,^35^ 50 μM Isorhamnetin,^16^ 20 μM Calycosin,^36^ 20 μM Kaempferol^37^ and 20 μM Formononetin^38^). All the compounds were purchased from Sigma‐Aldrich.

### 2.8 Immunofluorescent (IF) staining

Cells were rinsed thrice in PBS before being fixed in 4% paraformaldehyde for 15 minutes, washed in PBS, and incubated for 20 min at ambient temperature in 0.5% Triton X‐100 before being rinsed in PBS. Dropwise injections of bovine serum albumin (5%) were used to the petri dishes, followed by incubation for 30 min at ambient temperature. Next, the cells were incubated in anti‐α‐actinin at 4°C overnight (#3134, Cell Signalling, Danvers, MA, USA), washed, incubated for 30 min at 37°C in the buffer of FITC‐conjugated goat anti‐rabbit IgG away from the light, rinsed with PBS, stained in 6‐diamidino‐2‐phenylindole (DAPI) and examined under (Olympus, Tokyo, Japan).

### 2.9 qRT‐PCR

RNA was extracted from H9C2 cells using RNeasy Mini Kit (Qiagen). Next, cDNA was synthesized and subjected to quantitative PCR using SYBR Premix Ex TaqTM (Takara, Tokyo, Japan) and certain primers to examine the expression levels of TNF‐α, IL‐1β, IL‐18, IL‐6, ANP, BNP, β‐MHC and CTGF.

### 2.10 Western blot assay

Total protein of H9C2 cells were extracted applying RIPA lysate (Beyotime, Shanghai, China). After quantitative analysis using the bicinchoninic acid method, 80 μg total proteins were segregated using SDS‐polyacrylamide gel electrophoresis and transported onto polyvinylidene difluoride (PVDF) membranes. TBS‐T buffer supplementing 5% nonfat milk was served to block membranes for 1 h. Then the membranes experienced dispose with primary antibodies including anti‐ESR1 antibody (AF6058, 1/1000, Affinity Biosciences, Changzhou, China) and anti‐ Phospho‐ESR1 antibody (AF3058, 1/1000, Affinity Biosciences) at 4°C overnight. After rinsed employing TBST thrice, the membranes were following blended with the horseradish peroxidase (HRP)‐linked secondary antibody goat anti‐rabbit IgG H&L (ab6721, 1/2000, Abcam, Cambridge, MA, USA) at 25°C for 1.5 h. Signal detection was implemented with an ECL system (Life technologies corporation, Gaithersburg, MD, USA).

### 2.11 Enzyme‐linked immunosorbent assay (ELISA)

ELISA kits of TNF‐α (CSB‐E11987r, CUSABIO, Wuhan, China), IL‐1β (E‐EL‐R0012c, Elabscience, Wuhan, China), IL‐18 (E‐EL‐R0567c, Elabscience), IL‐6 (CSB‐E04640r, CUSABIO) and ESR1 (CSB‐E06848r, CUSABIO) were employed to detect TNF‐α, IL‐1β, IL‐18 and IL‐6 levels in the medium of H92c cells and ESR1 levels in H92c cells according the manufacture's instructions.

### 2.12 Statistical analyses

The cell experiments were performed in triplicate biological replicates. GraphPad Prism software 8 (GraphPad Software, Inc. La Jolla, CA, USA) was employed to analyse data. Comparisons between groups were assessed using a Student's *t*‐test. Comparisons among groups were assessed using one‐way analysis of variance (ANOVA) followed by Tukey's post‐test. The results are expressed as the means ± standard error of the mean (SEM). The significance level was set at *p* < 0.05.

### 2.13 Results the molecular structure, biological activity, and target types of HQ active compounds

A total of 15 active ingredients of HQ were obtained based on TCMSP (Table S1); among them, Isorhamnetin, Quercetin, Calycosin, Formononetin, and Kaempferol were found to be linked to heart failure (HF_related). The molecular structures, biological activity types, and target types of these five active compounds were shown in Figure S1: Isorhamnetin (Figure S1A), Quercetin (Figure S1B), Calycosin (Figure S1C), Formononetin (Figure S1D) and Kaempferol (Figure S1E). A total of 4010 drug targets of the active ingredients in HQ were obtained (Table S2).

### 2.14 KEGG signalling pathway enrichment annotation analysis on differentially expressed genes

**FIGURE 1:**
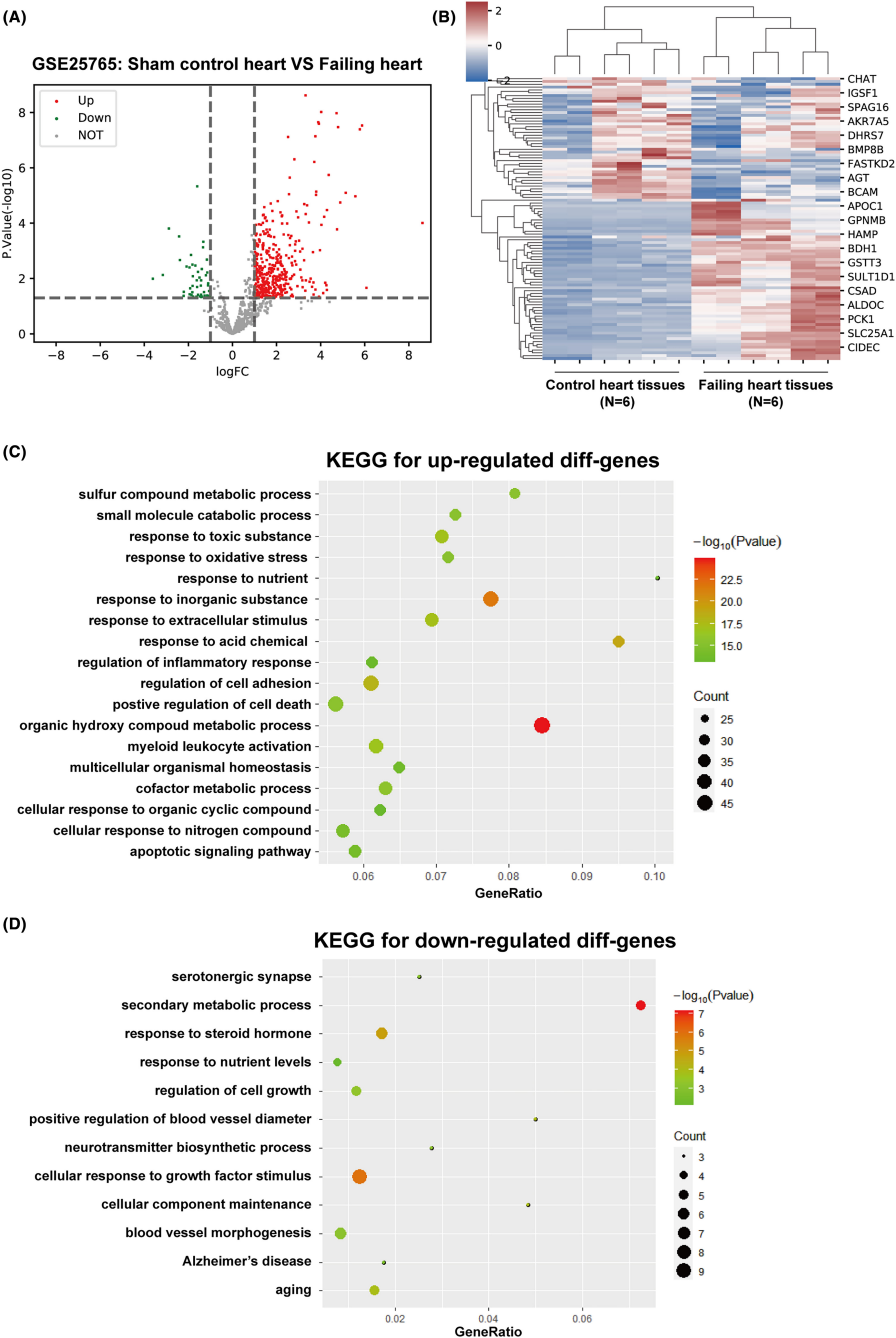
Kyoto Encyclopaedia of Genes and Genomes (KEGG) signalling pathway enrichment annotation analysis on differentially expressed genes. (A, B) Differentially expressed genes in heart tissue from failing hearts relative to healthy heart tissue, according to GSE25765. (C, D) Kyoto Encyclopaedia of Genes and Genomes (KEGG) signalling pathway enrichment annotation was performed on up‐ and down‐regulated genes obtained from the last step.

**FIGURE 2:**
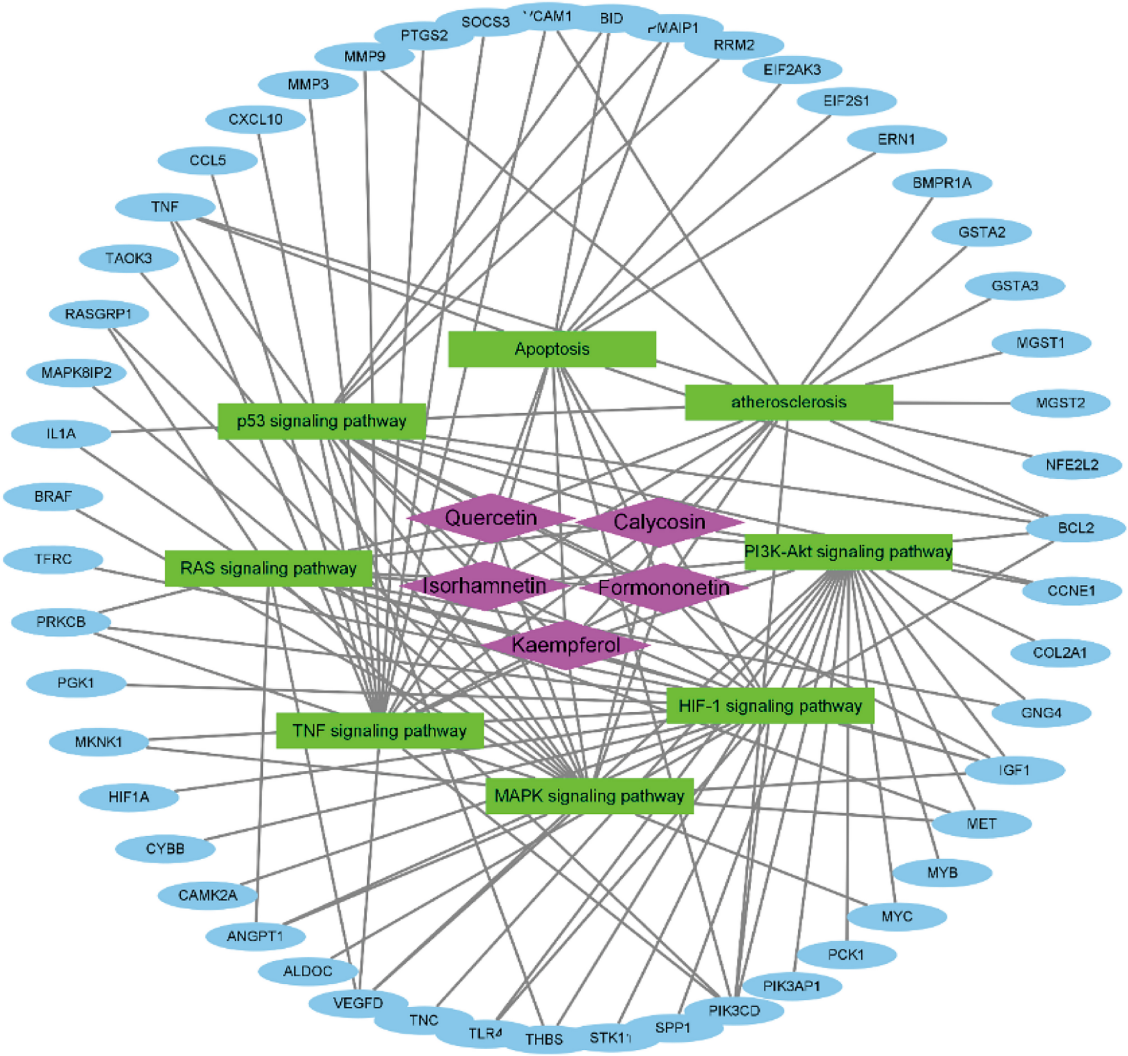
The compound‐signalling‐target networks generated using Cytoscape_v3.8.0. The compounds, pathways, or targeted proteins were represented by nodes in these graphical networks, whereas the interactions between compounds, pathways, or targeted proteins were represented by edges.

For identifying overlapped drug and disease targets, disease targets were retrieved by analysing differentially expressed genes. Among 4010 drug targets, a total of 391 genes were significant difference (348 up‐regulated and 43 down‐regulated) according to GSE25765 (Figure 1A,B). Up‐ and down‐regulated genes were applied for KEGG signalling pathway enrichment annotation respectively. Up‐regulated genes in failing hearts saw a significant enrichment in organic hydroxyl compounds and others' metabolic processes, cell apoptosis, adhesion ability, inflammatory response (Figure 1C). Down‐regulated genes in failing hearts saw a significant enrichment in secondary metabolism, the regulation of vascular diameter, steroid hormone response, cell growth and senescence (Figure 1D). The mechanism of differential genes in biological activities related to cardiovascular disease was shown in Figure S2, where the upregulated genes are represented by green blocks and the downregulated genes are represented by red blocks. Next, the compound‐signalling‐target networks between five active compounds, KEGG signalling pathway and differentially expressed genes were constructed (Figure 2, Table S3).

### 2.15 Effects of five active compounds on Ang II‐induced cardiomyocyte hypertrophy

**FIGURE 3:**
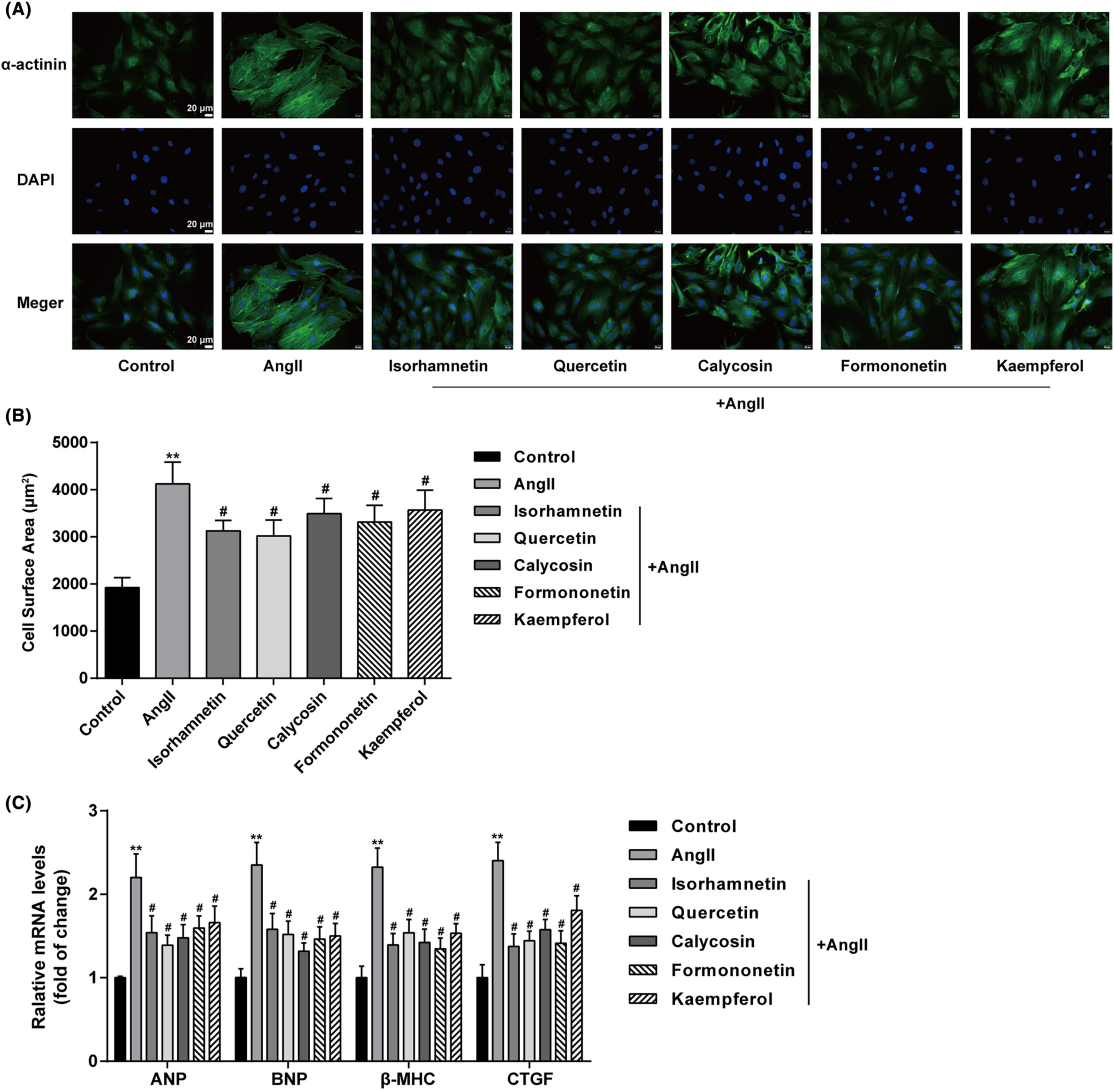
Effects of five active compounds on Ang II‐induced cardiomyocyte hypertrophy Cardiomyocytes were treated with Ang II (1 μM) for 24 h in the presence or absence of Quercetin (20 μM), Isorhamnetin (50 μM), Calycosin (20 μM), Kaempferol (20 μM), or Formononetin (20 μM) and examined for the content and distribution of α‐actinin by Immunofluorescent (IF) staining (A); cell surface area (B); the relative mRNA expression levels of ANP, BNP, β‐MHC, and CTGF by qRT‐PCR assay (C). N = 3 (biological replicates). **p < 0.01 compared to control group; ^#^ *p* < 0.05 compared to Ang II group.

For investigating the specific effects of these five active compounds on cardiomyocyte hypertrophy, cardiomyocytes were treated with 1 μM Ang II for 24 h with or without Quercetin, Isorhamnetin, Calycosin, Kaempferol, or Formononetin. As the marker of cardiomyocyte hypertrophy, α‐actinin saw a significant increase in cardiomyocytes stimulated by Ang II alone; Ang II‐induced increase in α‐actinin was partially eliminated by Quercetin, Isorhamnetin, Calycosin, Kaempferol, or Formononetin (Figure 3A). Cell surface areas were calculated accordingly; consistent with the morphological appearance, Ang II significantly enlarged the cell surface area, which could be partially reduced by Quercetin, Isorhamnetin, Calycosin, Kaempferol, or Formononetin (Figure 3B). Finally, Ang II significantly upregulated ANP, BNP, β‐MHC mRNA expression, and CTGF, whereas Quercetin, Isorhamnetin, Calycosin, Kaempferol, or Formononetin treatment partially downregulated the expression levels of ANP, BNP, β‐MHC and CTGF (Figure 3C).

### 2.16 Five active compounds of *Astragalus membranaceus* attenuated Ang II‐induced inflammation in cardiomyocytes

**FIGURE 4:**
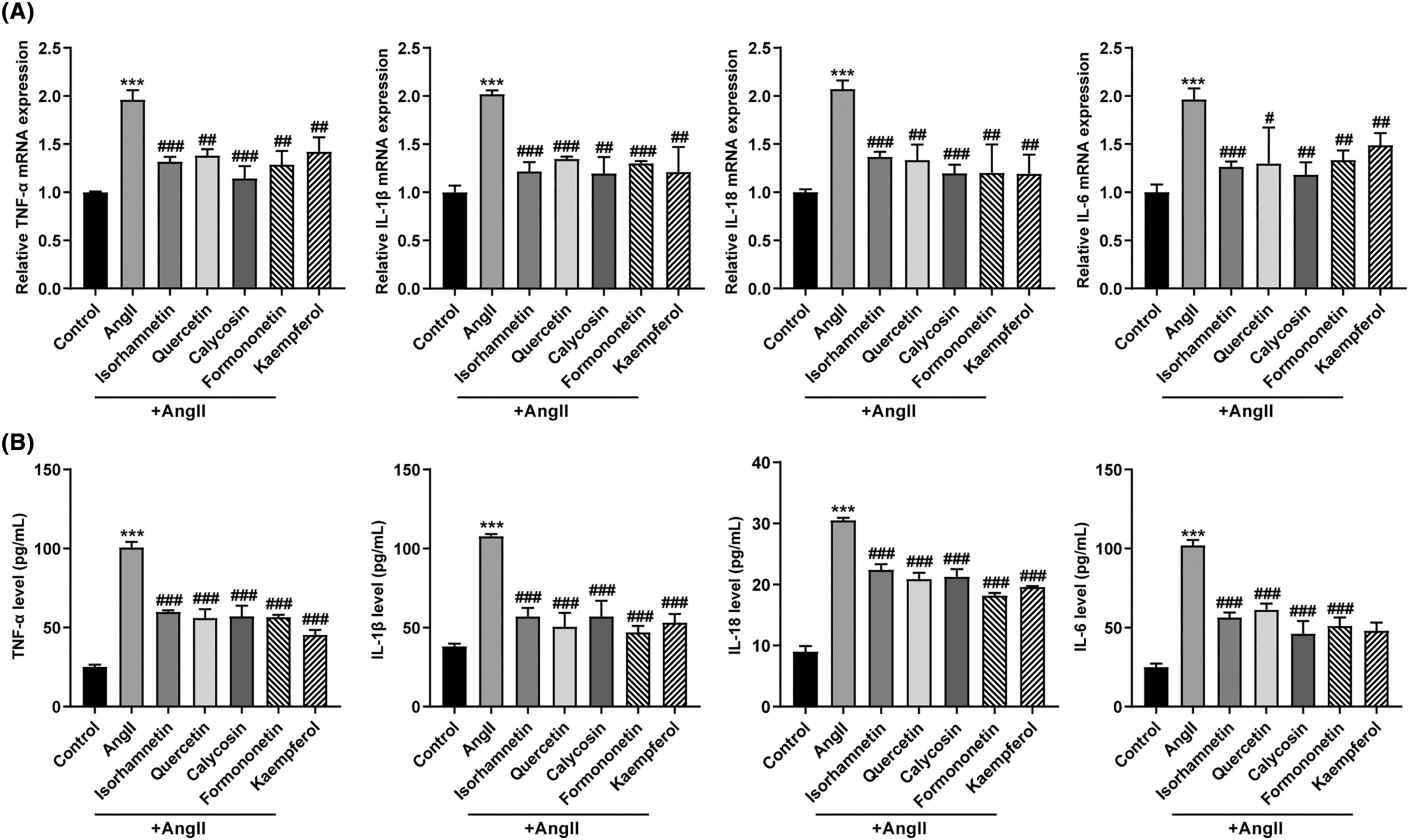
Effects of five active compounds of *Astragalus membranaceus* on Ang II‐induced proinflammatory cytokine release in cardiomyocytes. Cardiomyocytes were treated with Ang II (1 μM) for 24 h in the presence or absence of Quercetin (20 μM), Isorhamnetin (50 μM), Calycosin (20 μM), Kaempferol (20 μM), or Formononetin (20 μM) and then examined for the mRNA levels of TNF‐α, IL‐1β, IL‐18 and IL‐6 in cardiomyocytes by qRT‐PCR assay (A); the levels of TNF‐α, IL‐1β, IL‐18 and IL‐6 in cardiomyocytes medium by ELISA assay (B). N = 3 (biological replicates). ***p < 0.001 compared to control group; ^#^ *p* < 0.05, ^##^ *p* < 0.01, ^###^ *p* < 0.001 compared to Ang II group.

Unresolved inflammation is a key mediator of advanced heart failure. Accordingly, the effects of these five active compounds on Ang II‐induced proinflammatory cytokine release in cardiomyocytes were investigated. qRT‐PCR (Figure 4A) and ELISA (Figure 4B) assays results showed Ang II increased the levels of proinflammatory cytokines including TNF‐α, IL‐1β, IL‐18 and IL‐6 in cardiomyocytes and the medium of cardiomyocytes, respectively. While Ang II‐induced increase in TNF‐α, IL‐1β, IL‐18 and IL‐6 was partially eliminated by Quercetin, Isorhamnetin, Calycosin, Kaempferol or Formononetin treatment (Figure 4A,B).

### 2.17 Prediction of the binding between HQ active ingredients and target genes by Molecular docking

**FIGURE 5:**
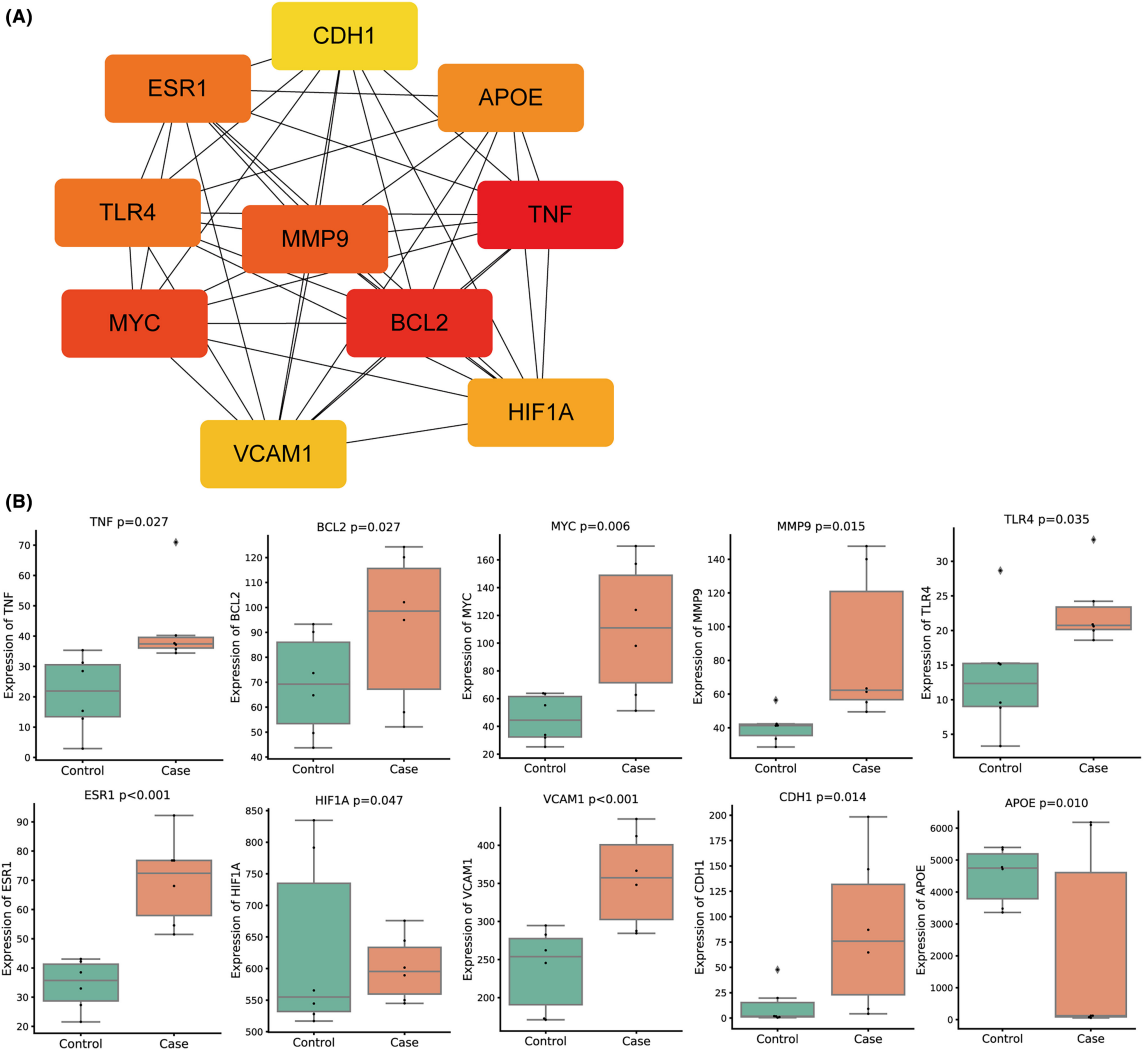
Screening and expression analysis of hub genes. (A) 391 drug genes were analysed, and a protein interaction network was constructed based on the online tool STRING database, with the thresholds of the interaction score greater than 0.9. Finally, 10 hub genes were screened. (B) Significant differences of 10 hub genes between failing heart tissues and control heart tissues were analysed based on GSE25765 database.

A total of 391 drug genes were analysed and constructed into the protein interaction networks. Finally, 10 hub genes were screened (Figure 5A). Next, significant differences of 10 hub genes between failing heart tissues and control heart tissues were analysed based on GSE25765 database. As indicated by the results, there was 1 differentially downregulated drug target gene (APOE), and 9 differentially upregulated drug target genes (TNF, BCL2, MYC, MMP9, TLR4, ESR1, HIF1A, VCAM1 and CDH1) in heart failure (Figure 5B).

**FIGURE 6:**
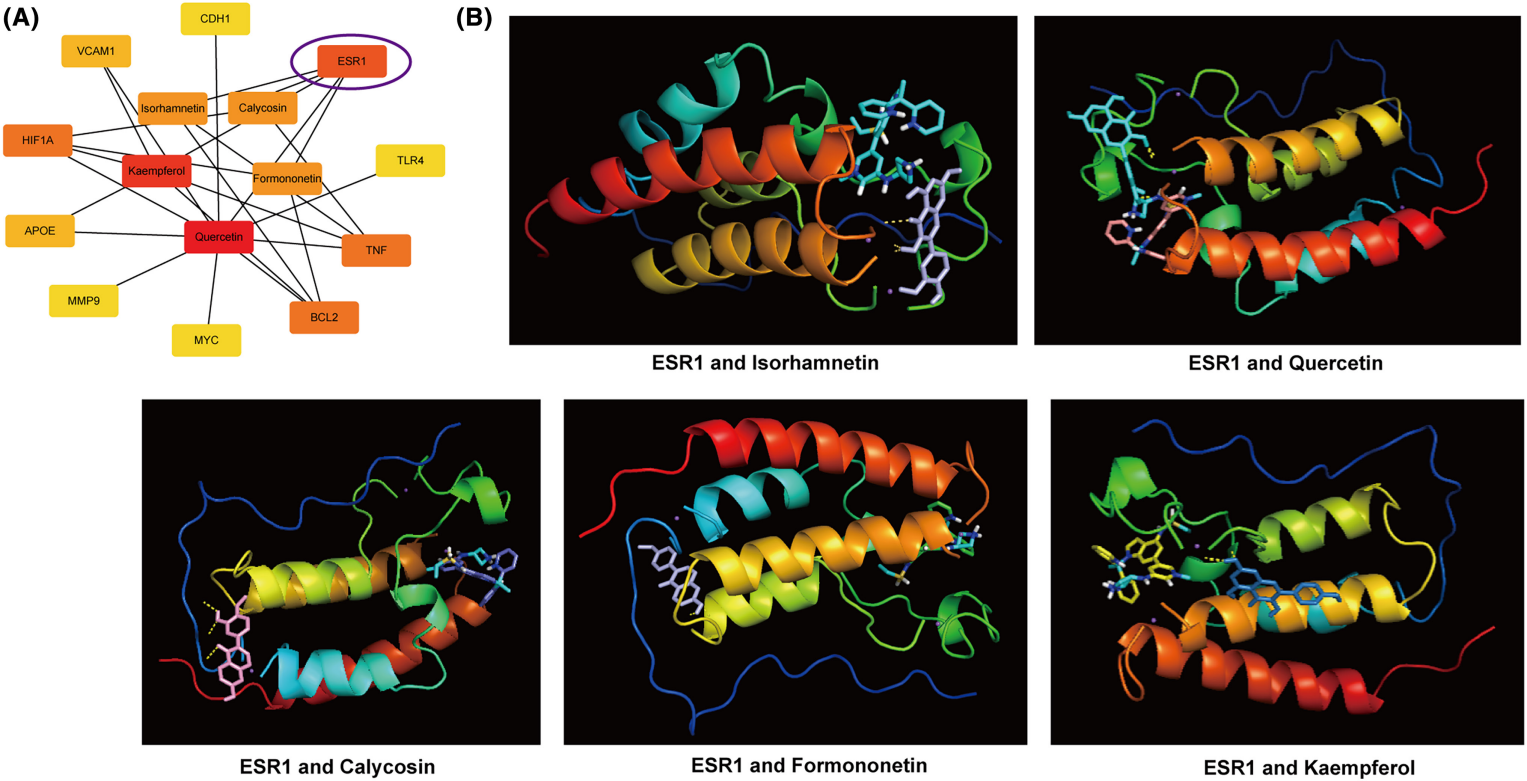
The docking model of isorhamnetin, quercetin, calycosin, formononetin, and kaempferol with ESR1. (A) The network diagram of 10 hub genes and 5 HQ active ingredients was constructed. (B) Molecular docking map of 5 HQ active ingredients and ESR1.

Then, the network diagram of 10 hub genes and 5 HQ active ingredients was constructed (Figure 6A). Among then, ESR1 is the common target gene of the five active ingredients. Previous studies have shown ESR1 might serve as a prognostic, diagnostic biomarker and therapeutic target for HF.^39^ Moreover, molecular docking was applied to verify the correlation between ESR1 and 5 HQ active ingredients (Figure 6B). The binding energy between ESR1 and Isorhamnetin, Quercetin, Calycosin, Formononetin or Kaempferol were −6.53, −7.53, −7.48, −8.26 or −6.92 kcal/mol, respectively. Together, five active ingredients (Quercetin, Isorhamnetin, Calycosin, Kaempferol and Formononetin) of HQ could bind well with a core target of HF (ESR1), all of which might play key roles in the treatment of HF.

### 2.18 Effects of five active compounds on ESR1 level in Ang II‐induced cardiomyocytes

**FIGURE 7:**
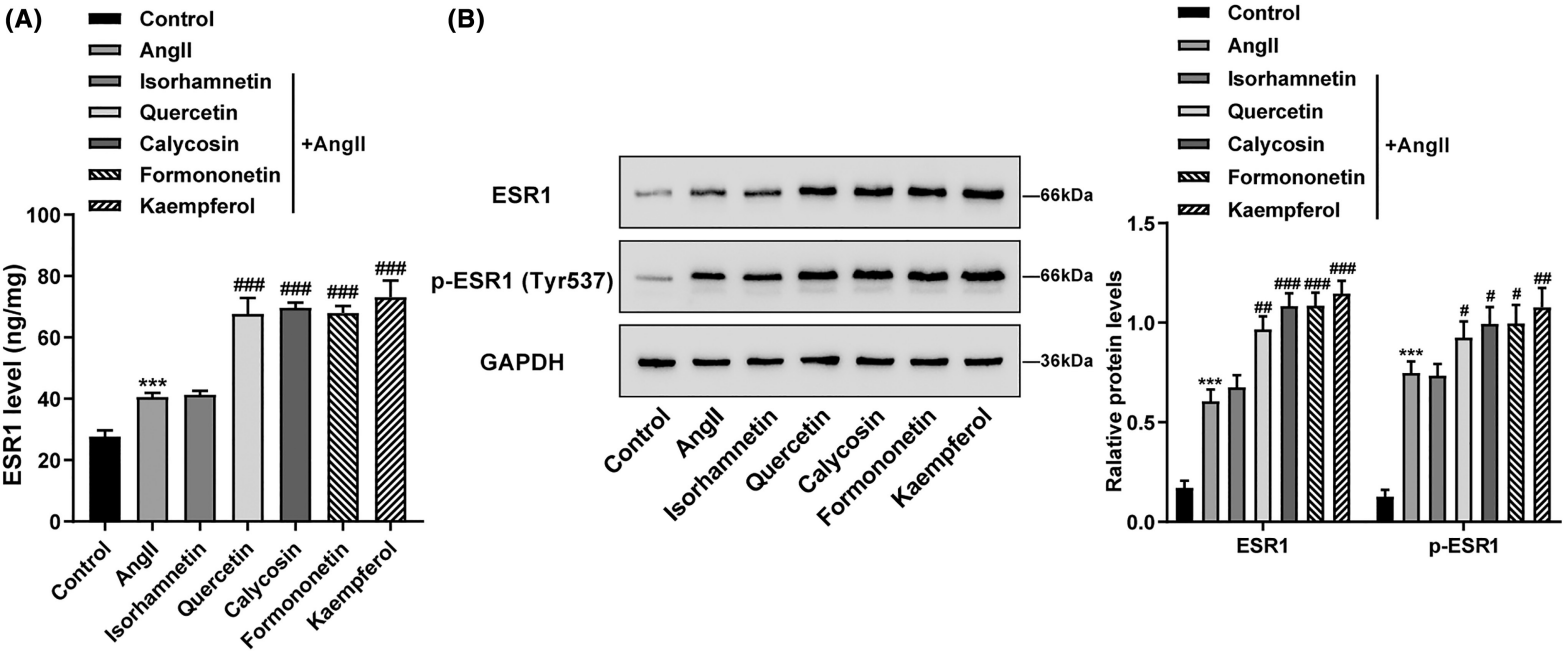
Effects of five active compounds on ESR1 level in Ang II‐induced cardiomyocytes. Cardiomyocytes were treated with Ang II (1 μM) for 24 h in the presence or absence of Quercetin (20 μM), Isorhamnetin (50 μM), Calycosin (20 μM), Kaempferol (20 μM), or Formononetin (20 μM) and then examined for the ESR1 level in cardiomyocytes by ELISA assay (A); the protein levels of ESR1 and phosphorylated‐ESR1 in cardiomyocytes by western blot assay (B). N = 3 (biological replicates). ***p < 0.001 compared to control group; ^#^ *p* < 0.05, ^##^ *p* < 0.01, ^###^ *p* < 0.001 compared to Ang II group.

Finally, the effects of these five active compounds on ESR1 expression in Ang II‐induced cardiomyocytes were investigated. As the results of qRT‐PCR (Figure 7A) and ELISA (Figure 7B) assays shown, the ESR1 and phosphorylated ESR1 expression levels were significantly increased in cardiomyocytes stimulated by Ang II, and Quercetin, Calycosin, Kaempferol or Formononetin treatment further promoted the expression levels of ESR1 and phosphorylated ESR1; however, Isorhamnetin treatment had no effect on ESR1 and phosphorylated ESR1 expression levels (Figure 7A,B). These outcomes concluded that Quercetin, Calycosin, Kaempferol and Formononetin could inhibit Ang II‐induced cardiomyocyte hypertrophy by promoting ESR1 expression.

## 3 DISCUSSION

The primary six syndromes in heart failure patients are deficiency of Yang, Qi, blood stasis, fluid retention, Yin deficiency, and phlegm turbidity.^40^, ^41^ Deficiency of Qi and blood will cause tissue energy, urine, and bodily fluid to stagnate.^42^ HQ, a tonic herb having ‘Gan flavour’ and ‘Wen nature’, is often used for benefiting ‘Qi’ and nourishing ‘blood’.^42^ HQ is capable of improving cardiovascular function, protecting myocardial cells, increasing coronary blood flow, enhancing cardiac contractility, and exerting positive inotropic action upon the heart.^43^, ^44^ The effect of HQ in the treatment of heart failure has been verified in a variety of animal models with the protective effects involve improving myocardial contraction, protecting myocardial cells, regulating the neuroendocrine system and inhibiting left ventricular remodelling.^15^, ^45^ In this study, a total of 15 active ingredients of HQ were obtained; among them, Isorhamnetin, Quercetin, Calycosin, Formononetin, and Kaempferol were found to be linked to heart failure (HF_related). Among 4010 drug targets, a total of 391 genes were with significant difference (348 up‐regulated and 43 down‐regulated). Up‐regulated genes saw a significant enrichment in cell apoptosis, adhesion ability, and inflammatory response. Down‐regulated genes saw a significant enrichment in secondary metabolism, the regulation of vascular diameter, steroid hormone response, and cell growth and senescence.

Considering previous findings of the therapeutic effects of Quercetin, Isorhamnetin, Calycosin, Kaempferol, and Formononetin of HQ on cardiovascular diseases and integrative bioinformatics analysis results, these five active compounds might dominate the cardioprotective effects of HQ. Then, for investigating the specific effects of the five main active compounds, Ang II‐induced cardiomyocyte hypertrophy model was exploited. Ang II induces an inflammatory phenotype in cardiomyocytes,^46^ and leads to cellular hypertrophy^47^ and increased deposition of matrix proteins.^48^ Furthermore, inhibiting Ang II has been shown to prevent cardiac hypertrophy and fibrosis.^49^ In this study, Ang II stimulation alone caused significant increase in cell size. In the meantime, the mRNA expression levels of cardiac function markers, such as ANP, BNP, β‐MHC and CTGF were significantly up‐regulated by Ang II treatment. ANP and BNP are expressed in the heart and are secreted in response to atrial and ventricular volume expansion, respectively.^50^, ^51^ Up‐regulated ANP, BNP, β‐MHC, and α‐skeletal actin could be observed in pathological cardiac hypertrophy.^52^ CTGF, a downstream mediator of the profibrotic functions of TGF‐β pathway, is a secreted matricellular protein acting in concert with TGF‐β1 to cause fibrogenesis.^53^ Therefore, Ang II stimulation induced cardiomyocyte hypertrophy and fibrogenesis. Notably, when treated with Isorhamnetin, Quercetin, Calycosin, Formononetin or Kaempferol, Ang II‐induced increases in cell size and ANP, BNP, β‐MHC and CTGF expression levels were partially decreased, suggesting that these five main active compounds could reverse Ang II‐induced cardiomyocyte hypertrophy and fibrogenesis.

Moreover, molecular docking revealed five active ingredients (Quercetin, Isorhamnetin, Calycosin, Kaempferol and Formononetin) of HQ may could bind well with a core target (ESR1) of HF. Oestrogen receptors are involved in the development of cardiovascular disease.^54^ Oestrogen receptor 1 (ESR1) encodes an oestrogen receptor and ligand‐activated transcription factor. The ESR1 genetic variant is related to the susceptibility of coronary heart disease.^55^ The increase of total ESR1 expression contributes to the stability of cardiac intercalated discs.^56^ ESR1 transcriptional pathway is essential to couple cellular energy metabolism with energy consumption processes in order to maintain normal cardiac function.^57^ Moreover, the congestive heart failure is associated with a significant decrease of myocardial ESR1.^58^ ESR1 might serve as a prognostic, diagnostic biomarker and therapeutic target for HF.^39^ Here, we found the ESR1 and phosphorylated ESR1 expression levels were significantly increased in cardiomyocytes stimulated by Ang II, and Quercetin, Calycosin, Kaempferol, or Formononetin treatment further promoted the expression levels of ESR1 and phosphorylated ESR1; however, Isorhamnetin treatment had no effect on ESR1 and phosphorylated ESR1 expression levels. Previously, García‐Gutiérrez et al. demonstrated that Quercetin significantly increased the expression of ESR1 in HT‐29 colon cancer cells.^59^ Calycosin promoted the expression of ER‐α36, which is the main oestrogen receptor of ESR1, in macrophages.^60^ Kaempferol notably promoted ESR1 expression in the ovaries tissue of rats with premature ovarian failure.^61^ In HUVECs, MCF‐7 and BT474 cells, Formononetin markedly up‐regulated ESR1 expression.^62^ All these outcomes uncovered that four active ingredients (Quercetin, Calycosin, Kaempferol and Formononetin) of HQ could bind well with a core target (ESR1) of HF, all of which play key roles through regulating ESR1 expression in the treatment of HF.

In conclusion, Isorhamnetin, Quercetin, Calycosin, Formononetin, and Kaempferol might be the primary active ingredients of HQ, dominating its cardioprotective effects against heart failure probably through regulating ESR1 expression, which provided a potential mechanism for the clinical application of HQ to regulate cardiac hypertrophy and heart failure.

## AUTHOR CONTRIBUTIONS

**Qiuxiang Chen:** Conceptualization (equal); writing – original draft (equal). **Juan Wang:** Data curation (equal); resources (equal); software (equal). **Lihua Sun:** Validation (equal); visualization (equal). **Bayinsilema Ba:** Formal analysis (equal); methodology (equal). **Difei Shen:** Project administration (lead); writing – review and editing (lead).

## FUNDING INFORMATION

This study was supported by National Natural Science Foundation of China (No. 81960052 and 81660039) and Xinjiang Medical University scientific research innovation team project (XYD2024C11).

## CONFLICT OF INTEREST STATEMENT

The authors confirm that there are no conflicts of interest.

## CONSENT FOR PUBLICATION

Not applicable.

## Supporting information


**Figure S1.** The molecular structure, biological activity, and target types of *Astragalus membranaceus* (Huangqi, HQ) active compounds, Isorhamnetin (A), Quercetin (B), Calycosin (C), Formononetin (D), and Kaempferol (E).

**Figure S2.** Enriched signalling pathways.

**Table S1.** The main active ingredient of *Astragalus membranaceus* in TCMSP datebase (OB≥40%, DL≥0.2).

**Table S2.** The function and drug target gene of these 5 active components.

**Table S3.** Compound‐signalling‐target networks of the five active ingredients of HQ.

## Data Availability

Please contact the authors for data requests.

## References

[1] Tomasoni D , Adamo M , Lombardi CM , Metra M . Highlights in heart failure. ESC Heart Fail. 2019;6(6):1105‐1127. doi:10.1002/ehf2.12555 31997538 PMC6989277

[2] Baman JR , Ahmad FS . Heart failure. JAMA. 2020;324(10):1015. doi:10.1001/jama.2020.13310 32749448

[3] Mercurio V , Pirozzi F , Lazzarini E , et al. Models of heart failure based on the cardiotoxicity of anticancer drugs. J Card Fail. 2016;22(6):449‐458. doi:10.1016/j.cardfail.2016.04.008 27103426

[4] Anjos M , Fontes‐Oliveira M , Costa VM , Santos M , Ferreira R . An update of the molecular mechanisms underlying doxorubicin plus trastuzumab induced cardiotoxicity. Life Sci. 2021;280:119760. doi:10.1016/j.lfs.2021.119760 34166713

[5] Quagliariello V , Berretta M , Buccolo S , et al. Polydatin reduces cardiotoxicity and enhances the anticancer effects of sunitinib by decreasing pro‐oxidative stress, pro‐inflammatory cytokines, and NLRP3 inflammasome expression. Front Oncol. 2021;11:680758. doi:10.3389/fonc.2021.680758 34178667 PMC8226180

[6] Tham YK , Bernardo BC , Ooi JYY , Weeks KL , McMullen JR . Pathophysiology of cardiac hypertrophy and heart failure: signaling pathways and novel therapeutic targets. Arch Toxicol. 2015;89(9):1401‐1438. doi:10.1007/s00204-015-1477-x 25708889

[7] Rosca MG , Tandler B , Hoppel CL . Mitochondria in cardiac hypertrophy and heart failure. J Mol Cell Cardiol. 2013;55:31‐41. doi:10.1016/j.yjmcc.2012.09.002 22982369 PMC3805050

[8] Li X‐Q , He J‐C , Huang P‐X , Cao X‐B . Chinese medicine syndromes in congestive heart failure: a literature study and retrospective analysis of clinical cases. Chin J Integr Med. 2016;22(10):738‐744. doi:10.1007/s11655-015-2085-6 26906719

[9] Wang Y , Wang Q , Li C , et al. A review of Chinese herbal medicine for the treatment of chronic heart failure. Curr Pharm Des. 2017;23(34):5115‐5124. doi:10.2174/1381612823666170925163427 28950815 PMC6340156

[10] Bi Y‐F , Mao J‐Y , Wang X‐L , Wang H‐H , Ge Y‐B , Zhang Z‐P . Contemporary treatment of Western and Chinese medicine for cardiac syndrome X. Chin J Integr Med. 2011;17(4):314‐320. doi:10.1007/s11655-011-0714-2 21509677

[11] Liang D , Zhang M . The thinking on TCM differential treatment of congestive heart failure. J Tradit Chin Med. 2000;20(1):44‐47. 10921172

[12] Liu P , Zhao H , Luo Y . Anti‐aging implications of astragalus Membranaceus (Huangqi): a well‐known Chinese tonic. Aging Dis. 2017;8(6):868‐886. doi:10.14336/AD.2017.0816 29344421 PMC5758356

[13] Feng W , Yang J , Li Y , Sun H , Zhang J , Xue Y . Astragaloside IV alleviates heart failure by modulating Nrf‐2. Chin Med J. 2022;135(9):1099‐1101. doi:10.1097/CM9.0000000000001828 34743151 PMC9276071

[14] Ma D , Wu T , Qu Y , et al. Astragalus polysaccharide prevents heart failure‐induced cachexia by alleviating excessive adipose expenditure in white and brown adipose tissue. Lipids Health Dis. 2023;22(1):9. doi:10.1186/s12944-022-01770-3 36670439 PMC9863193

[15] Fu S , Zhang J , Menniti‐Ippolito F , et al. Huangqi injection (a traditional Chinese patent medicine) for chronic heart failure: a systematic review. PLoS One. 2011;6(5):e19604. doi:10.1371/journal.pone.0019604 21573109 PMC3089614

[16] Gao L , Yao R , Liu Y , et al. Isorhamnetin protects against cardiac hypertrophy through blocking PI3K‐AKT pathway. Mol Cell Biochem. 2017;429(1–2):167‐177. doi:10.1007/s11010-017-2944-x 28176246

[17] Li Y , Xu C , Wang H , et al. Systems pharmacology reveals the multi‐level synergetic mechanism of action of Ginkgo biloba L. leaves for cardiomyopathy treatment. J Ethnopharmacol. 2021;264:113279. doi:10.1016/j.jep.2020.113279 32810617

[18] Zhao DD , Zhang XQ , Yang T , et al. Exploring the therapeutic mechanism of Tingli Dazao Xiefei decoction on heart failure based on network pharmacology and experimental study. Evid Based Complement Alternat Med. 2021;2021:6645878. doi:10.1155/2021/6645878 34868332 PMC8639272

[19] Aonuma K , Ferdousi F , Xu D , Tominaga K , Isoda H . Effects of isorhamnetin in human amniotic epithelial stem cells in vitro and its cardioprotective effects in vivo. Front Cell Dev Biol. 2020;8:578197. doi:10.3389/fcell.2020.578197 33117805 PMC7552739

[20] Chang X , Zhang T , Meng Q , et al. Quercetin improves cardiomyocyte vulnerability to hypoxia by regulating SIRT1/TMBIM6‐related mitophagy and endoplasmic reticulum stress. Oxidative Med Cell Longev. 2021;2021:5529913. doi:10.1155/2021/5529913 PMC8024107 33859776

[21] Wang L , Tan A , An X , Xia Y , Xie Y . Quercetin dihydrate inhibition of cardiac fibrosis induced by angiotensin II in vivo and in vitro. Biomed Pharmacother. 2020;127:110205. doi:10.1016/j.biopha.2020.110205 32403046

[22] Zhang F , Zhang Y , Li X , et al. Research on Q‐markers of Qiliqiangxin capsule for chronic heart failure treatment based on pharmacokinetics and pharmacodynamics association. Phytomedicine. 2018;44:220‐230. doi:10.1016/j.phymed.2018.03.003 29699844

[23] He L , Liu Y , Yang K , et al. The discovery of Q‐markers of Qiliqiangxin capsule, a traditional Chinese medicine prescription in the treatment of chronic heart failure, based on a novel strategy of multi‐dimensional "radar chart" mode evaluation. Phytomedicine. 2021;82:153443. doi:10.1016/j.phymed.2020.153443 33429210

[24] Yun WJ , Yao ZH , Fan CL , et al. Systematic screening and characterization of Qi‐Li‐Qiang‐Xin capsule‐related xenobiotics in rats by ultra‐performance liquid chromatography coupled with quadrupole time‐of‐flight tandem mass spectrometry. J Chromatogr B Analyt Technol Biomed Life Sci. 2018;1090:56‐64. doi:10.1016/j.jchromb.2018.05.014 29787993

[25] Huang C , Qiu S , Fan X , et al. Evaluation of the effect of Shengxian decoction on doxorubicin‐induced chronic heart failure model rats and a multicomponent comparative pharmacokinetic study after oral administration in normal and model rats. Biomed Pharmacother. 2021;144:112354. doi:10.1016/j.biopha.2021.112354 34794233

[26] Wang J , Fang X , Ge L , et al. Antitumor, antioxidant and anti‐inflammatory activities of kaempferol and its corresponding glycosides and the enzymatic preparation of kaempferol. PLoS One. 2018;13(5):e0197563. doi:10.1371/journal.pone.0197563 29771951 PMC5957424

[27] Calderon‐Montano JM , Burgos‐Moron E , Perez‐Guerrero C , Lopez‐Lazaro M . A review on the dietary flavonoid kaempferol. Mini Rev Med Chem. 2011;11(4):298‐344. doi:10.2174/138955711795305335 21428901

[28] Du Y , Han J , Zhang H , Xu J , Jiang L , Ge W . Kaempferol prevents against ang II‐induced cardiac remodeling through attenuating ang II‐induced inflammation and oxidative stress. J Cardiovasc Pharmacol. 2019;74(4):326‐335. doi:10.1097/FJC.0000000000000713 31356553 PMC6791499

[29] Hopkins AL . Network pharmacology: the next paradigm in drug discovery. Nat Chem Biol. 2008;4(11):682‐690. doi:10.1038/nchembio.118 18936753

[30] Ru J , Li P , Wang J , et al. TCMSP: a database of systems pharmacology for drug discovery from herbal medicines. J Chem. 2014;6:13. doi:10.1186/1758-2946-6-13 PMC4001360 24735618

[31] Zeng X , Zhang P , He W , et al. NPASS: natural product activity and species source database for natural product research, discovery and tool development. Nucleic Acids Res. 2018;46(D1):D1217‐D1222. doi:10.1093/nar/gkx1026 29106619 PMC5753227

[32] Kanehisa M , Sato Y , Kawashima M . KEGG mapping tools for uncovering hidden features in biological data. Protein Sci. 2021;31:47‐53. doi:10.1002/pro.4172 34423492 PMC8740838

[33] Smoot ME , Ono K , Ruscheinski J , Wang PL , Ideker T . Cytoscape 2.8: new features for data integration and network visualization. Bioinformatics. 2011;27(3):431‐432. doi:10.1093/bioinformatics/btq675 21149340 PMC3031041

[34] Eberhardt J , Santos‐Martins D , Tillack AF , Forli S . AutoDock Vina 1.2.0: new docking methods, expanded force field, and python bindings. J Chem Inf Model. 2021;61(8):3891‐3898. doi:10.1021/acs.jcim.1c00203 34278794 PMC10683950

[35] Chen K , Rekep M , Wei W , et al. Quercetin prevents in vivo and in vitro myocardial hypertrophy through the proteasome‐GSK‐3 pathway. Cardiovasc Drugs Ther. 2018;32(1):5‐21. doi:10.1007/s10557-018-6771-4 29435775

[36] Liu B , Zhang J , Liu W , et al. Calycosin inhibits oxidative stress‐induced cardiomyocyte apoptosis via activating estrogen receptor‐alpha/beta. Bioorg Med Chem Lett. 2016;26(1):181‐185. doi:10.1016/j.bmcl.2015.11.005 26620254

[37] Ma C , Xia R , Yang S , et al. Formononetin attenuates atherosclerosis via regulating interaction between KLF4 and SRA in apoE(−/−) mice. Theranostics. 2020;10(3):1090‐1106. doi:10.7150/thno.38115 31938053 PMC6956811

[38] Liu Y , Gao L , Guo S , et al. Kaempferol alleviates angiotensin II‐induced cardiac dysfunction and interstitial fibrosis in mice. Cell Physiol Biochem. 2017;43(6):2253‐2263. doi:10.1159/000484304 29073623

[39] Kolur V , Vastrad B , Vastrad C , Kotturshetti S , Tengli A . Identification of candidate biomarkers and therapeutic agents for heart failure by bioinformatics analysis. BMC Cardiovasc Disord. 2021;21(1):329. doi:10.1186/s12872-021-02146-8 34218797 PMC8256614

[40] Chen C , Meng YM , Zhang P , et al. Diagnosis and treatment rule of traditional Chinese medicine for syndrome factors of chronic congestive heart failure: a study based on Shannon entropy method. Zhong Xi Yi Jie He Xue Bao. 2010;8(11):1080‐1084. doi:10.3736/jcim20101113 21078274

[41] Shao‐Mei W , Li‐Fang Y , Li‐Hong W . Traditional Chinese medicine enhances myocardial metabolism during heart failure. Biomed Pharmacother. 2022;146:112538. doi:10.1016/j.biopha.2021.112538 34922111

[42] Yue SJ , Liu J , Feng WW , et al. System pharmacology‐based dissection of the synergistic mechanism of Huangqi and Huanglian for diabetes mellitus. Front Pharmacol. 2017;8:694. doi:10.3389/fphar.2017.00694 29051733 PMC5633780

[43] Yu J , Zhang X , Zhang Y . Astragaloside attenuates myocardial injury in a rat model of acute myocardial infarction by upregulating hypoxia inducible factor1alpha and Notch1/Jagged1 signaling. Mol Med Rep. 2017;15(6):4015‐4020. doi:10.3892/mmr.2017.6522 28487976 PMC5436283

[44] Han R , Tang F , Lu M , et al. Astragalus polysaccharide ameliorates H_2_O_2_‐induced human umbilical vein endothelial cell injury. Mol Med Rep. 2017;15(6):4027‐4034. doi:10.3892/mmr.2017.6515 28487940 PMC5436204

[45] Liu Y , Xu W , Xiong Y , Du G , Qin X . Evaluations of the effect of HuangQi against heart failure based on comprehensive echocardiography index and metabonomics. Phytomedicine. 2018;50:205‐212. doi:10.1016/j.phymed.2018.04.027 30466980

[46] Valente AJ , Clark RA , Siddesha JM , Siebenlist U , Chandrasekar B . CIKS (Act1 or TRAF3IP2) mediates angiotensin‐II‐induced Interleukin‐18 expression, and Nox2‐dependent cardiomyocyte hypertrophy. J Mol Cell Cardiol. 2012;53(1):113‐124. doi:10.1016/j.yjmcc.2012.04.009 22575763 PMC3477707

[47] Guo H , Liu B , Hou L , et al. The role of mAKAPbeta in the process of cardiomyocyte hypertrophy induced by angiotensin II. Int J Mol Med. 2015;35(5):1159‐1168. doi:10.3892/ijmm.2015.2119 25739102 PMC4380120

[48] Singh VP , Le B , Khode R , Baker KM , Kumar R . Intracellular angiotensin II production in diabetic rats is correlated with cardiomyocyte apoptosis, oxidative stress, and cardiac fibrosis. Diabetes. 2008;57(12):3297‐3306. doi:10.2337/db08-0805 18829990 PMC2584136

[49] Dai DF , Johnson SC , Villarin JJ , et al. Mitochondrial oxidative stress mediates angiotensin II‐induced cardiac hypertrophy and Galphaq overexpression‐induced heart failure. Circ Res. 2011;108(7):837‐846. doi:10.1161/CIRCRESAHA.110.232306 21311045 PMC3785241

[50] Edwards BS , Zimmerman RS , Schwab TR , Heublein DM , Burnett JC Jr . Atrial stretch, not pressure, is the principal determinant controlling the acute release of atrial natriuretic factor. Circ Res. 1988;62(2):191‐195. doi:10.1161/01.res.62.2.191 2962782

[51] Kinnunen P , Vuolteenaho O , Ruskoaho H . Mechanisms of atrial and brain natriuretic peptide release from rat ventricular myocardium: effect of stretching. Endocrinology. 1993;132(5):1961‐1970. doi:10.1210/endo.132.5.8477647 8477647

[52] Bernardo BC , Weeks KL , Pretorius L , McMullen JR . Molecular distinction between physiological and pathological cardiac hypertrophy: experimental findings and therapeutic strategies. Pharmacol Ther. 2010;128(1):191‐227. doi:10.1016/j.pharmthera.2010.04.005 20438756

[53] Abreu JG , Ketpura NI , Reversade B , De Robertis EM . Connective‐tissue growth factor (CTGF) modulates cell signalling by BMP and TGF‐beta. Nat Cell Biol. 2002;4(8):599‐604. doi:10.1038/ncb826 12134160 PMC2387275

[54] Aryan L , Younessi D , Zargari M , et al. The role of estrogen receptors in cardiovascular disease. Int J Mol Sci. 2020;21(12):4314. doi:10.3390/ijms21124314 32560398 PMC7352426

[55] Sun Y , Cheng Z , Cui M , et al. GAS5/METTL14/ESR1 genetic variants are related to the susceptibility of coronary heart disease. Funct Integr Genomics. 2022;22(3):341‐357. doi:10.1007/s10142-022-00831-1 35235104

[56] Mahmoodzadeh S , Eder S , Nordmeyer J , et al. Estrogen receptor alpha up‐regulation and redistribution in human heart failure. FASEB J. 2006;20(7):926‐934. 16675850 10.1096/fj.05-5148com

[57] Wang T , McDonald C , Petrenko NB , et al. Estrogen‐related receptor α (ERRα) and ERRγ are essential coordinators of cardiac metabolism and function. Mol Cell Biol. 2015;35(7):1281‐1298. doi:10.1128/MCB.01156-14 25624346 PMC4355525

[58] Hu X , Xu X , Lu Z , et al. AMP activated protein kinase‐α2 regulates expression of estrogen‐related receptor‐α, a metabolic transcription factor related to heart failure development. Hypertension. 2011;58(4):696‐703. doi:10.1161/HYPERTENSIONAHA.111.174128 21825219 PMC3182261

[59] García‐Gutiérrez N , Luna‐Bárcenas G , Herrera‐Hernández G , et al. Quercetin and its fermented extract as a potential inhibitor of bisphenol A‐exposed HT‐29 colon cancer Cells' viability. Int J Mol Sci. 2023;24(6):5604. doi:10.3390/ijms24065604 36982678 PMC10052295

[60] Wu G , Qi G , Liu Y , et al. ER‐α36 is involved in calycosin inhibition of IL‐6 production in macrophages. J Cell Mol Med. 2023;28:e18037. doi:10.1111/jcmm.18037 37974543 PMC10805506

[61] Liu H , Yang H , Qin Z , et al. Exploration of the Danggui Buxue decoction mechanism regulating the balance of ESR and AR in the TP53‐AKT signaling pathway in the prevention and treatment of POF. Evid Based Complement Alternat Med. 2021;2021:4862164. doi:10.1155/2021/4862164 35003302 PMC8739177

[62] Chen J , Zhang X , Wang Y , Ye Y , Huang Z . Differential ability of formononetin to stimulate proliferation of endothelial cells and breast cancer cells via a feedback loop involving MicroRNA‐375, RASD1, and ERα. Mol Carcinog. 2018;57(7):817‐830. doi:10.1002/mc.22531 29722068

