# Supplementary figures and images for: Mechanism of Astragalus membranaceus (Huangqi, HQ) for treatment of heart failure based on network pharmacology and molecular docking
Source: J Cell Mol Med. 2024 May 23;28(10):e18331. doi: 10.1111/jcmm.18331 (PMC11114218; doi:10.1111/jcmm.18331)

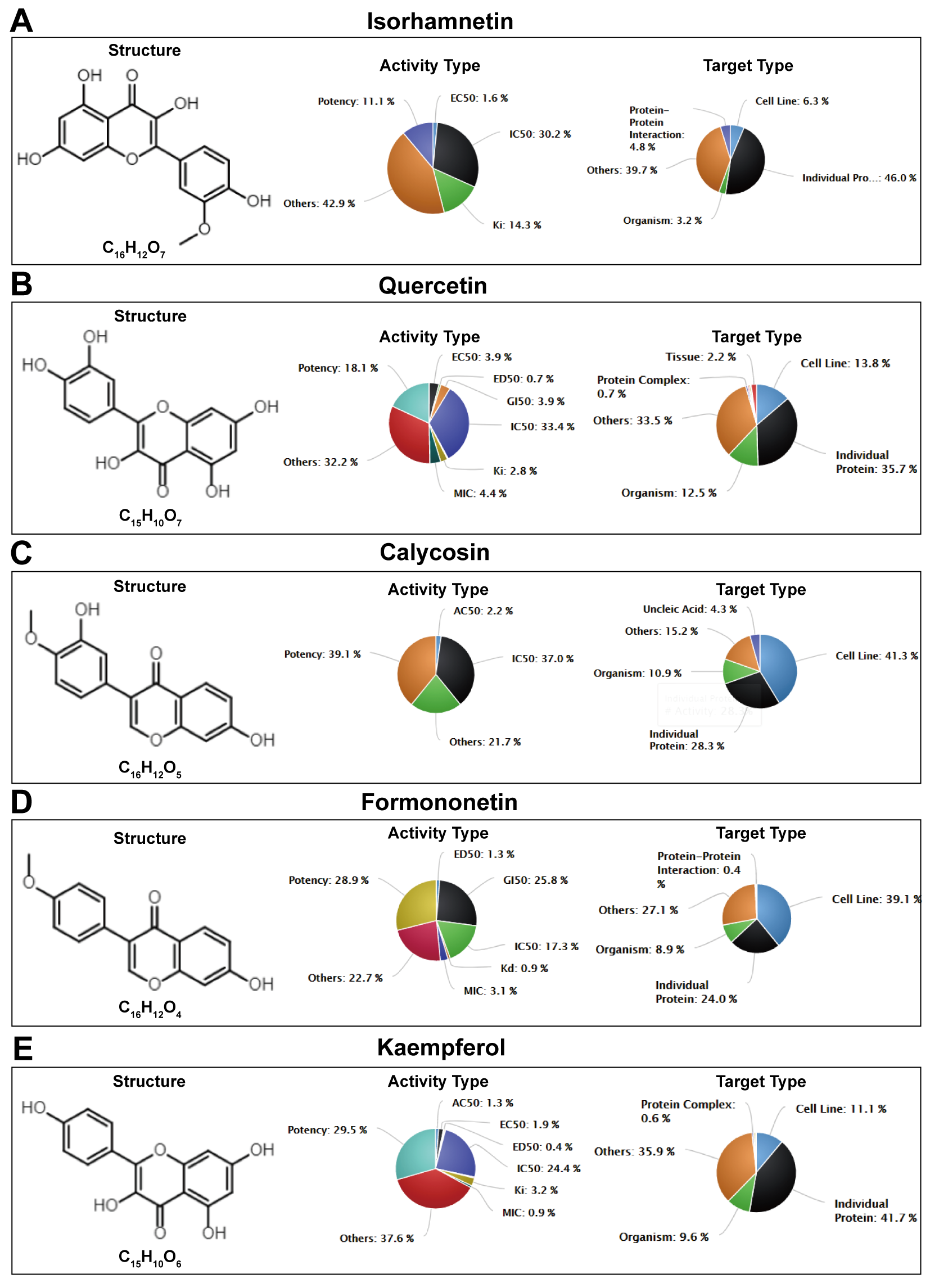

Supplement: Supplementary file 1 — Figure S1. The molecular structure, biological activity, and target types of Astragalus membranaceus (Huangqi, HQ) active compounds, Isorhamnetin (A), Quercetin (B), Calycosin (C), Formononetin (D), and Kaempferol (E). [file JCMM-28-e18331-s003.tif]

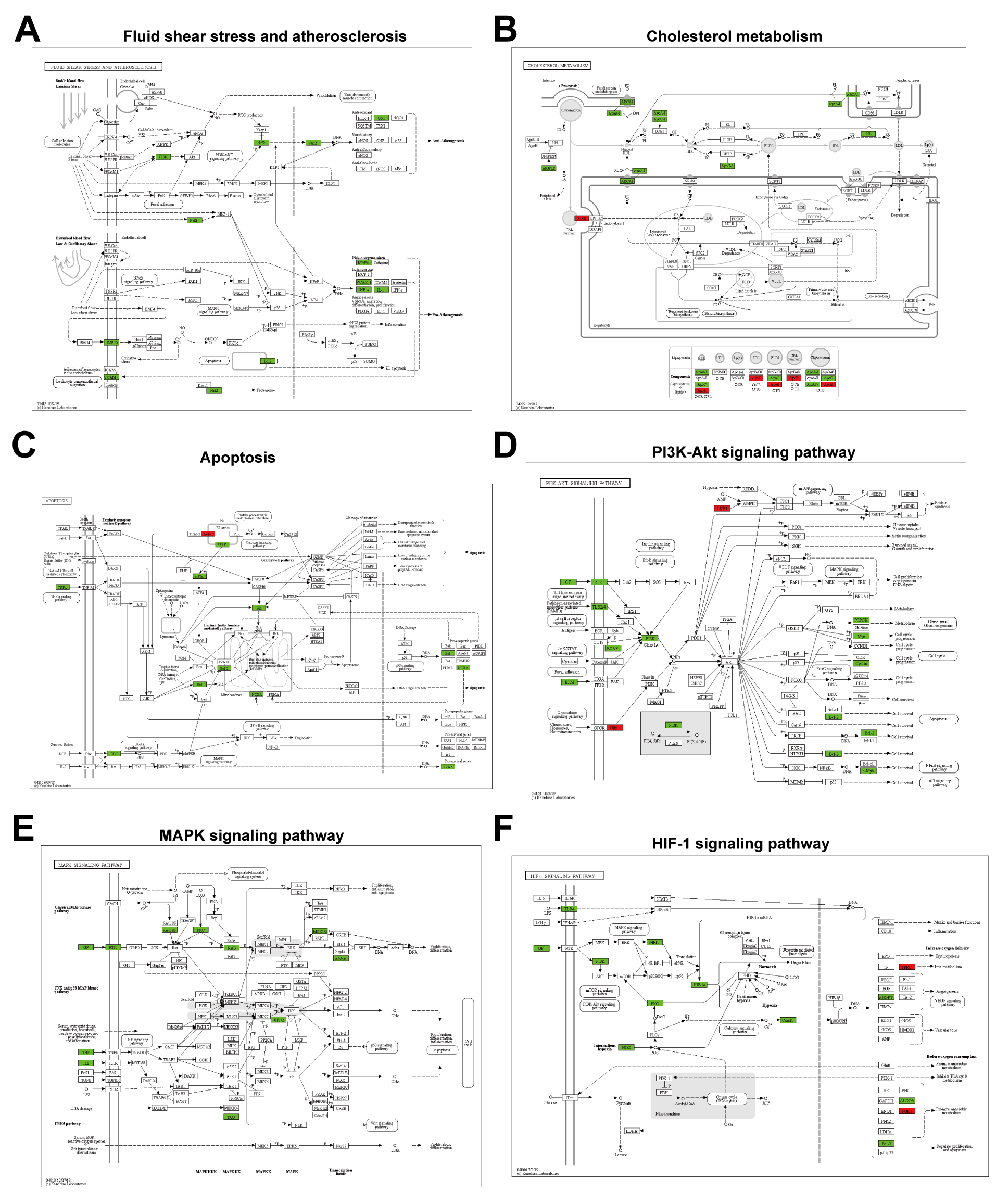

Supplement: Supplementary file 2 — Figure S2. Enriched signalling pathways. [file JCMM-28-e18331-s005.tif]
