# Supplementary material for: Mechanism of Astragalus membranaceus (Huangqi, HQ) for treatment of heart failure based on network pharmacology and molecular docking
Source: J Cell Mol Med. 2024 May 23;28(10):e18331. doi: 10.1111/jcmm.18331 (PMC11114218; doi:10.1111/jcmm.18331)
Supplement: Supplementary file 4 — Table S2. The function and drug target gene of these 5 active components. [file JCMM-28-e18331-s001.doc]

**Table S2. The function and drug target gene of these 5 active components**

| **Molecule Name** | **Signal pathway** | [**Function**](../../../../C:%5CProgram%20Files%20(x86)%5CYoudao%5CDict%5C7.2.0.0703%5Cresultui%5Cdict%5C%3Fkeyword=function) | **Number of target genes** | **source** | | |
| --- | --- | --- | --- | --- | --- | --- |
|  |  |  |  | DGIdb | CTD | DrugBank |
| Isorhamnetin | PI3K-Akt, MAPK, p53, IL-17, TNF | Cell senescence, atherosclerosis | 56 | 3 | 54 | 0 |
| Quercetin | PI3K-Akt, MAPK | Apoptosis, cell senescence, cytokine receptor action | 3969 | 79 | 3939 | 37 |
| Calycosin | TNF, IL-17, RAS, JAK/STAT | Type C lectin receptor signal | 57 | 3 | 54 | 0 |
| Kaempferol | MAPK, TNF, IL-17, RAS, AMPK, Chemokine | Atherosclerosis, apoptosis | 183 | 17 | 172 | 1 |
| Formononetin | PI3K-Akt, p53, IL-17 | Apoptosis | 44 | 3 | 41 | 0 |

**Note:** DGIdb, Drug Gene Interaction database, http://www.dgidb.org;

CTD, Comparative Toxicogenomics Database; http://ctdbase.org/;

DrugBank, DrugBank, http://www.drugbank.ca/.
