# Supplementary material for: Mechanism of Astragalus membranaceus (Huangqi, HQ) for treatment of heart failure based on network pharmacology and molecular docking
Source: J Cell Mol Med. 2024 May 23;28(10):e18331. doi: 10.1111/jcmm.18331 (PMC11114218; doi:10.1111/jcmm.18331)
Supplement: Supplementary file 5 — Table S3. Compound‐signalling‐target networks of the five active ingredients of HQ. [file JCMM-28-e18331-s004.doc]

**Table S3. compound-signaling-target networks of the five active ingredients** of HQ

| **Compound** | **Node(Target)** | **Edge(Pathway)** |
| --- | --- | --- |
| Quercetin | 51 | 8 |
| Isorhamnetin | 4 | 8 |
| Calycosin | 3 | 8 |
| Kaempferol | 10 | 8 |
| Formononetin | 2 | 8 |
